# The paradigm and future value of the metaverse for the intervention of cognitive decline

**DOI:** 10.3389/fpubh.2022.1016680

**Published:** 2022-10-20

**Authors:** Hao Zhou, Jian-Yi Gao, Ying Chen

**Affiliations:** ^1^Faculty of Science, The University of Sydney, Sydney, NSW, Australia; ^2^Institute of Medical Genetics, Nanjing Medical University Affiliated Wuxi Maternity and Child Health Care Hospital, Wuxi, China; ^3^Jiangnan University Affiliated Wuxi Maternity and Child Health Care Hospital, Wuxi, China

**Keywords:** mental health, cognitive decline, Alzheimer's disease, metaverse in medicine, virtual reality, digital twin

## Abstract

Cognitive decline is a gradual neurodegenerative process that is affected by genetic and environmental factors. The doctor-patient relationship in the healthcare for cognitive decline is in a “shallow” medical world. With the development of data science, virtual reality, artificial intelligence, and digital twin, the introduction of the concept of the metaverse in medicine has brought alternative and complementary strategies in the intervention of cognitive decline. This article technically analyzes the application scenarios and paradigms of the metaverse in medicine in the field of mental health, such as hospital management, diagnosis, prediction, prevention, rehabilitation, progression delay, assisting life, companionship, and supervision. The metaverse in medicine has made primary progress in education, immersive consultation, dental disease, and Parkinson's disease, bringing revolutionary prospects for non-pharmacological complementary treatment of cognitive decline and other mental problems. In particular, with the demand for non-face-to-face communication generated by the global COVID-19 epidemic, the needs for uncontactable healthcare service for the elderly have increased. The paradigm of self-monitoring, self-healing, and healthcare experienced by the elderly through the metaverse in medicine, especially from meta-platform, meta-community, and meta-hospital, will be generated, which will reconstruct the service modes for the elderly people. The future map of the metaverse in medicine is huge, which depends on the co-construction of community partners.

## Introduction

By 2050, the global population over the age of 60 will be about 210 million, and more than 130 million may suffer from Alzheimer's disease (AD) (1). AD is an aging-related disease characterized by progressive cognitive decline and cerebral cortex atrophy, leading to dementia. The etiology of cognitive decline is complex and affected by genetic and environmental factors. Many theories and hypotheses on Etiology, Neuropathology and Pathology have been proposed (2, 3). AD is usually too late to be discovered because of the occult features at the initial stage. Most drugs based on the amyloid cascade hypothesis failed at the trial stage (4), which led to no effective treatment after diagnosis. The alternative and complementary strategies of non-pharmacological prevention and intervention for people at risk or treatment for the patients need to be exploited.

At present, the relationship established between patients with cognitive decline and their doctors is still in a “shallow” medical world, and the service for the healthcare for cognitive decline needs to be improved. First, the ordinary medical archives did not fully cover the information about the patient's daily life and their environmental conditions. The diagnosis and treatment were based on insufficient participation of the patients lacking data on life logging. Second, scientific management in life, sports, and entertainment for the elderly was lacking. Third, trust and effective communication between doctors and patients were lacking. To effectively solve the above problems, establishing a new medical world with deep empathy among doctors and patients is urgent. This article technically analyzed the application scenarios and paradigms of the metaverse in mental health and proposed that people in the related fields around the world work together.

## What is the metaverse in medicine

Telehealthcare has been used in the treatment and research of intellectual developmental disorders (5), which provides a model for the intervention of cognitive decline using the technology of the metaverse. “Metaverse” is a three-dimensional (3D) internet application and digital social platform of the next generation with the essential features of an artificial virtual world (4). Introducing the framework of the metaverse into the intervention of cognitive decline is expected to reconstruct the service patterns for the elderly. In particular, during the global COVID-19 pandemic, the demands for non-face-to-face healthcare increased, and the metaverse expanded its application space.

**Figure 1:**
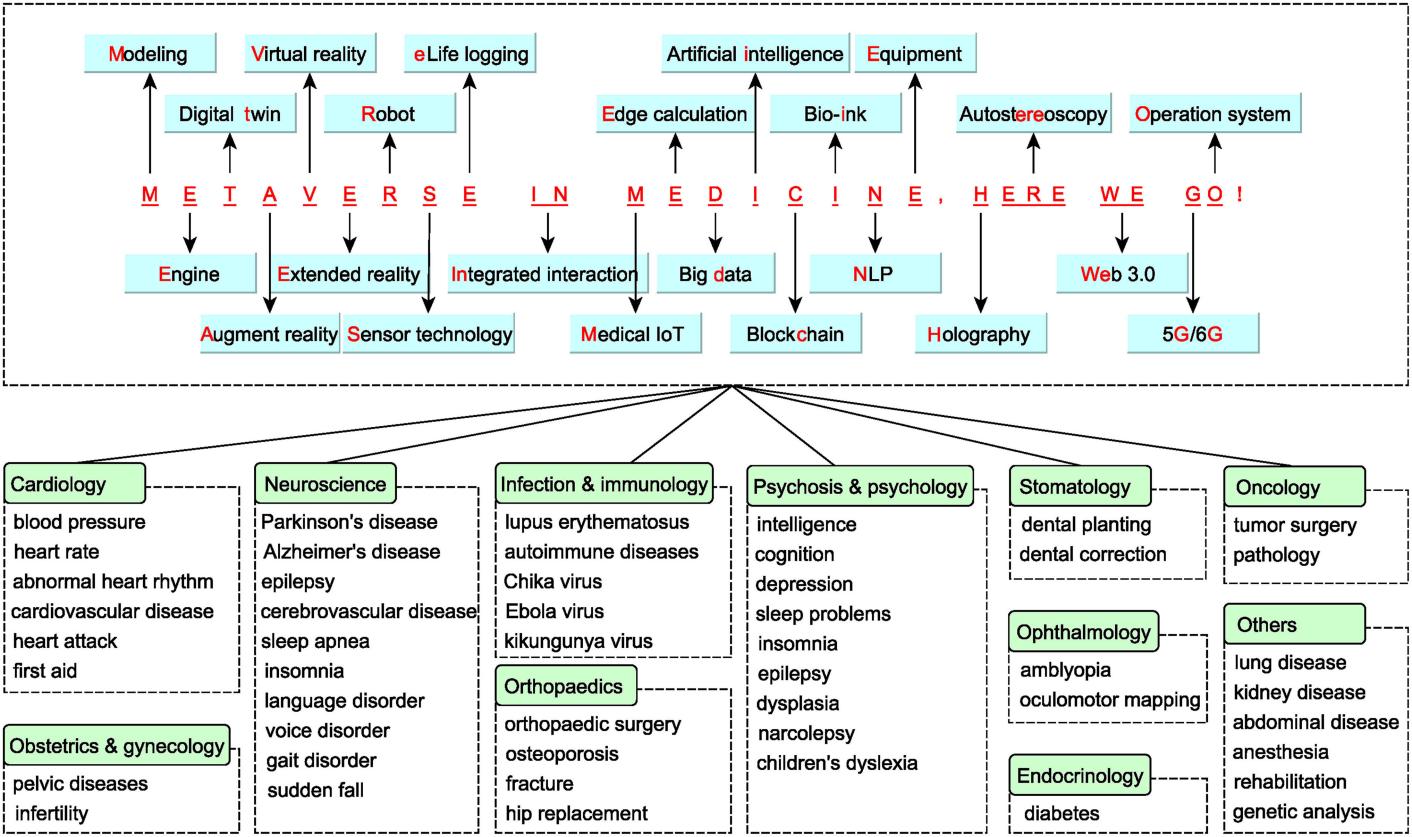
Medicine application of metaverse supporting technology in various fields. We concluded the main supporting technology of the metaverse in a sentence. They have been applied to different subjects and diseases. IoT, Internet of Things; NLP, natural language processing.

The metaverse technology applied in the field of education and teaching is called the Metaverse in Education, and applied in the field of healthcare and medicine is called the Metaverse in Health and the Metaverse in Medicine. The International Association and Alliance of Metaverse in Medicine (IAMM) released an Expert Consensus, defining 2022 as the 1st year of “Metaverse in Medicine,” and defining the Metaverse in Medicine as the medical internet of things (IOT) practiced through augmented reality (AR) technology using AR glasses or virtual reality (VR) glasses (6). Kye et al. (7) defined four types of the metaverse in medicine: AR, life logging, mirror world, and VR. Medical applications based on intelligent medicine, VR, and internet of things (IOT) application belong to the preform of the metaverse technology, which has penetrated into various medical fields and scenarios (8) (Figure 1). The metaverse in medicine supports immersive sense and has a wide range of application scenarios. It has made breakthroughs in medical education, training, medical license examination, immersive consultation, dental treatment, and Parkinson's disease treatment (9–12). The application of the metaverse in medicine is still in its infancy and has over 60 publications.

## Application scenarios of the metaverse in medicine in cognitive decline

The metaverse in medicine supports immersive senses and has a wide range of application scenarios. It can be applied in medical education and training, diagnosis, prediction, prevention, delay progress, supervision, elderly companionship, life assistance, and recovery for patients at risk or suffering from cognitive decline.

### Meta-hospital construction

Under the outbreak of COVID-19 pneumonia, the paradigm of non-face-to-face detection, self-healing, and self-healthcare will be generated through the metaverse platform, metaverse community, and metaverse hospital. “Meta-hospital” is a cloud platform of the metaverse in medicine serving cloud doctors, cloud patients, and educators, in which education, training, consultation, diagnosis and treatment, popular science publicity, home testing, and clinical research are carried out.

The digital twin technology in the metaverse is used to construct the digital scenarios of the hospital. A digital twin is a virtual form of a physical entity, with dynamic, bi-directional links between the physical entity and its original twin in the digital domain (13). Digital twins of people, goods, sites, equipment, systems, and even operation processes can be constructed and displayed from macro to micro dimensions in a multi-level and multi-fine-grained manner, which develop dynamically in parallel with the physical world. According to the requirements of the outpatient department, inpatient department, operating room, pharmacy, medical technology department, functional department, and supporting department, people and goods were positioned, tracked, and auto-transported in a 3D visible pattern. AR is to superimpose computer-generated virtual objects, scenarios, or annotations into the real physical world to enhance the presentation of reality. Complete mapping in virtual space by using physical models, sensor detection, operation history, and other data are built to realize a 3D visual intelligent hospital for full-time intelligent operation and maintenance. All the resources in the metaverse of medicine can be shared. Medical treatment, cloud diagnosis, cloud monitoring, and home testing can be carried out on populations online.

### Prediction and intervention using an algorithm

Artificial intelligence technology has obtained reliable results in genetic diagnosis and neuropsychological diagnoses of neurodevelopmental diseases, such as intellectual disability, autism, and depression (5, 14), which makes it possible to break through the bottleneck of early prediction and intervention of AD.

The clinical diagnosis of cognitive decline needs clinical evaluation and psychiatric test data, as well as neuroimaging and neurophysiological data. Brain neuroimaging and neurophysiological data provide important information on the changes in brain structure and function. In view of the gradual development of AD, full-time lifelogging, at-home motion sensor, multi-mode environment monitor, wearable monitor, and handwriting computer analysis were used to continuously record the activities, so as to obtain the dynamic data that could not be obtained before (15). Sensor (digital) data, handwriting (text), and voice (audio) data provided unique opportunities to identify new factors of cognitive decline (16, 17).

To realize the fusion of the above multimodal data, a transformer-based attention iteration model is introduced. In this model, data of different modes can be input. The input image is flattened into (m^*^c) dimensions, where m represents the size of pixel blocks ^*^ pixel blocks in the image, and c represents the number of pictures. The input text data are treated by natural language processing and recognized by the model in the form of (m^*^c) dimension. In the cross attention layer of the model, Q(uery), K(ey), and V(alue) are used to split the input vector into a series of < key, value > data pairs. By calculating the similarity and correlation between Q and each element, the weight coefficient of V corresponding to each k is obtained, and the weighted sum of V is calculated. Since the flattened image data have a large m value, to reduce the dimension, the dimension-reduced Q is obtained through the asymmetry projection, and then enters the attention layer, which greatly reduces the calculation time and training complexity. The obtained hidden features were input into the transformer to get the feature results, and this process is repeated for 48 layers, and then the average results and final feature judgments are obtained.

For personalized prevention of cognitive decline, the metaverse and algorithm-based platform designed for the prevention of cognitive decline can intervene at the early stage by training the brain, stimulating neural function, repairing memory damage, and delaying deterioration. The more communication among specific brain regions, the better the reasoning ability, which ascertains the ability of faster response and processing, so as to restore fluid intelligence and cognition. The platform with game elements has been used in the treatment of neuropsychological diseases, such as delaying cognitive decline, dredging post-traumatic stress disorder (PTSD) and depression, relieving pain, improving amblyopia, coping with traumatic brain injury, and Parkinson's disease. Consultation and treatment by the man-machine dialog of mental disorders were realized in 2015. VR is an efficient rehabilitation tool for stroke, Parkinson's disease, and other neurological diseases (5). The sports elements in the VR platform have benefits for the improvement of the cognitive abilities of elderly people. It is proved to be a successful method. Zhu et al. systematically reviewed the use of VR in the intervention of cognitive and motor functions of the elderly with mild cognitive impairment. Several aspects have been improved, such as overall cognition, attention, executive function, and memory (18).

### Companionship and assisted living

With the development of the metaverse in medicine, more products with the function of lifelogging and with games and sports elements for the elderly will enter their lives. By 2022, the number of connected wearable devices in the world is estimated to exceed 1.1 billion. The Vidicon revenue and auto-cameras are designed for patients with severe memory loss. All the images, sound around them, and experiences were captured and stored, so as to help them recall where they have been and what they have done at any time. AI intelligent speakers accompanied the elderly to chat. A social robot named Elli Q could gradually get to know the elderly and help them with online social networking and online games. An alcove immersive social software solved the problem of spatial distance and could experience the reality of offline social interaction. With VR and AI technology, all-around immersive global travel, shopping, fitting, and exploration can be experienced without leaving home. With the microchips implanted in the human brain, human consciousness was digitized. It is expected to be used for reconnecting the patient with the outside world who suffered from injury to the brain and nerves. Combined with nano-robot targeted therapy, brain-computer interface (BCI) will give more exciting medical application prospects.

The metaverse is the next stage of internet mode to break the limitation of the real world. The physical world, the spiritual world, and the knowledge world are connected, and the human-machine and the spirit-machine are combined to give the elderly unlimited super ability and freedom, and bloom their lives and realize their dreams again.

### Holographic intelligent control of emergency

Metaverse is used in some time-dependent first aid processes, such as sudden death, stroke, myocardial infarction, fall, shock, asthma, choking, epilepsy, acute abdomen, and other sudden diseases of the elderly. There are two main stages: the pre-hospital stage and the treatment stage in the hospital. All the processes can be mimicked in fine-grain by digital twin technology. The time flow and information flow of life of the patients can be intelligently replayed, helping to identify the abnormalities and respond quickly. Using digital twin technology, a twin script based on the real ambulance and emergency environment using various sensors was digitally reconstructed, which highly restored the details of emergency facilities and equipment, integrated rendering and seamless roaming. The digital pre-judgment in the pre-h stage is very important for deciding what level of rescue action the rescuers trigger in the second stage. According to the patient's condition, decide whether the first aid team is triggered and the hospital needs to make preparations during the transfer. Virtual services make the physical world more efficient, more convenient, more secure, and lower cost.

## How to establish the platform of the metaverse in medicine

**Figure 2:**
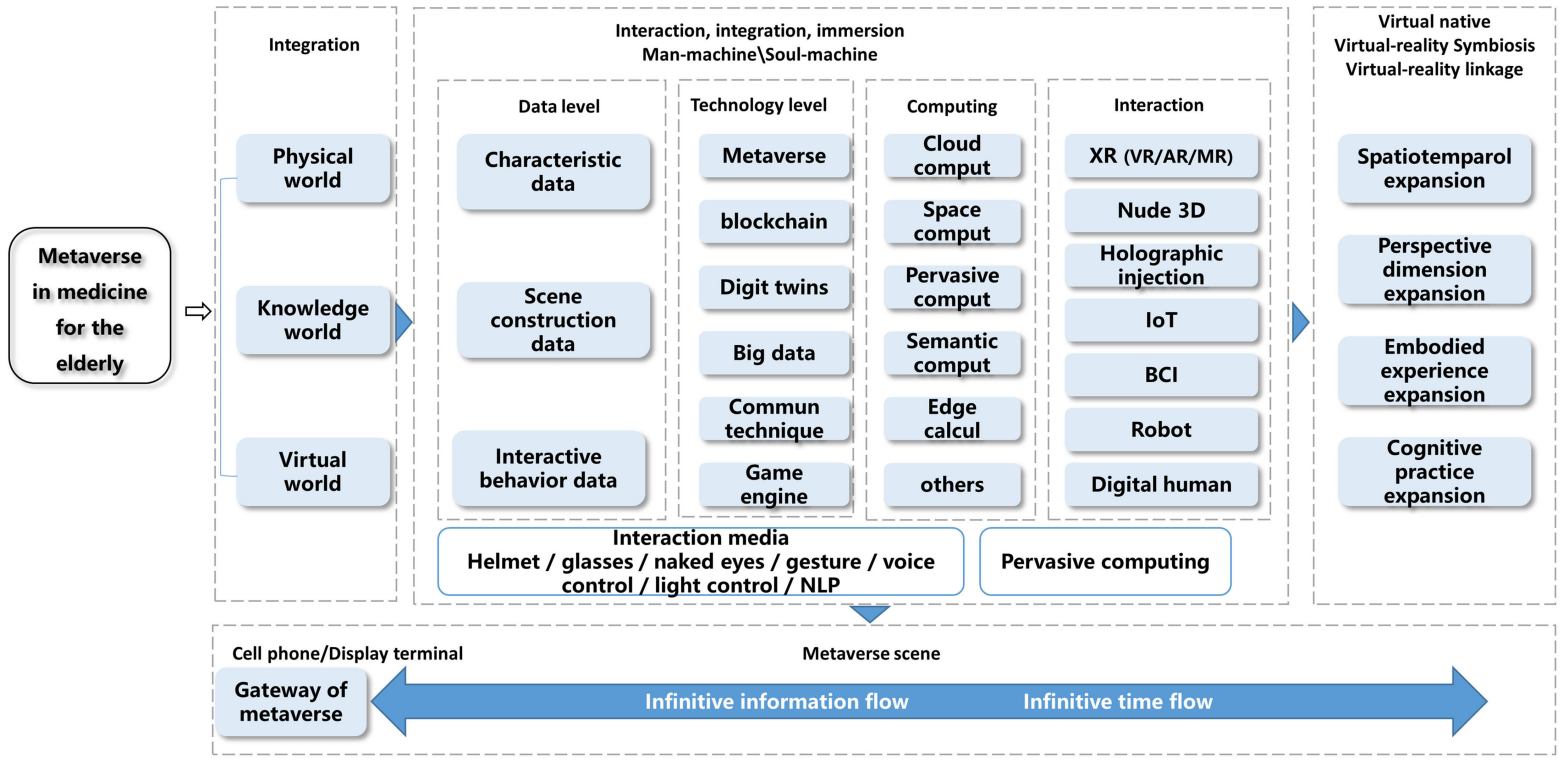
Diagram of technical stratification in realizing the metaverse in medicine for the elderly. IoT, internet of things; XR, extended reality; VR, virtual reality; AR, augmented reality; MR, mixed reality; BCI, brain computer interface; and NLP, natural language processing.

The metaverse requires the support of computing power and underlying technology, engine, platform, and APP. Among them, underlying technologies include digital twin, extended reality (XR), and internet of things (IoT) (as shown in Figure 2). The digital twin is used to generate the mirror digital space of the real world, which is the basis of the metaverse in medicine; extended reality (XR) is used to realize the immersive experience with 3D mixed scenarios, and construct the virtual and real connection at the human-computer interface. Internet of things (IOT) is used as the sensor entrance and the bottom technology of interaction.

Take the SINSO project as an example. SINSO is a decentralized medical infrastructure paradigm of open source (i.e., all program source codes are open). It was launched by the international cooperation team in 2020 and promoted in the community. The brief structure is as follows.

The project module includes four components: decentralized application (DAPP), gateway, donor networks, and distributed autonomous corporation (DAC). The SINSO DAPP component can quickly match the experts for consultation. Based on the smart contract of the distributed autonomous company (DAC), users uploaded data through the API interface of the Gateway component. The data were labeled and classified on the Gateway component and preprocessed to protect privacy. On the Donor networks component, users' data assets were converted into non-fungible token (NFT) assets and automatically matched the purchasers. Authorized inquirers find files through the address in the blockchain, using the public key and private key to generate encryption keys to re-encrypt the files and feed them back to the inquirer. Data sharing was realized and value flowed on the premise of maximizing privacy and data security. On the DAPP components, the members were continuously expanded and encouraged from pilot area to large-scale demo area, from many micro metaverse to decentralized medical infrastructure, such as metacommunity and meta hospital.

The periphery of SINO needs to be largely improved at this stage. A considerable part of the work is still similar to the process of road and bridge construction. Anonymous sharing of the medical data all over the world is a huge project for the medical industry, and the construction work is always on the road.

## Prospects and limitations

We tried to plan the foreseeable blueprint of the metaverse in medicine through the existing applications. Although the current technology has not kept up with the imagination, at least what we can predict is that the market demand and application scope will increase continuously with the maturity of the technology. The metaverse will stimulate medical innovation and upgrade in a new way. It will make medical diagnosis and treatment more accurate, fast, and convenient, and its position will develop from auxiliary to dependent and indispensable function. In particular, the metaverse will break the rule of geriatric diagnosis and treatment habits. The proportion of self-service and remote diagnosis and treatment will increase. The management role of hospitals will be weakened, and hospitals will become a distributed node and lose their central position.

On the other hand, because the world is facing uncertainty risks (COVID-19 variants, local conflicts, and economic inflation), in the short term, aging medicine is also subject to uncertainty pressures, such as shrinking internal demand, supply shocks, and weakening expectations, and the development and application of geriatric metaverse products are also restricted. Furthermore, the technology has its own limitations. For example, a 3D experience is not ideal since the digital twin is still at an early stage. The construction cost is high. The small metaverse established dispersedly without a unified standard will face problems when integrating later. The research and development of methods for multimodal technology integration lag behind. The XR equipment access to the metaverse is not convenient. With more and more clients accessing, the problems caused by large concurrent network connections need to be solved. Therefore, it needs to be deeply integrated with different industries to form a unified standard for sharing data, and finally can be integrated. Although the prospect is not very clear at present, in the long run, the metaverse is the way to the future of medicine.

## Conclusion

The world will be at the peak of aging. Long-term and unremitting efforts should be made to improve this social problem. It is necessary to take population-based AD prediction and mental healthcare as long-term goals. Fortunately, with the advent of population aging, we are entering the fourth industrial revolution triggered by AI technology, which brings hope to solve many aging-related problems. As an innovative medical environment, the metaverse in medicine represents a milestone of intelligent medicine. People from all fields actively seize positions in the metaverse. In particular, with the global epidemic, a growing need for uncontact medicine and healthcare for the elderly has emerged, which put forward the demand for non-face-to-face social communication. Through the platform of the metaverse in medicine, the elderly users will activate their own strength, reshape the elderly health system and lifestyle, and experience the paradigm of self-monitoring, self-diagnosis, self-healing, and self-care. It may help the elderly from a consumer group to a self-service group with the ability of sustainable development. As more data are added to the metaverse in medicine, this new paradigm will become an important preventive tool for the diagnosis and treatment of cognitive decline and AD.

## Author contributions

HZ is responsible for drafting some contents of AI and metaverse. J-YG is responsible for literature retrieval. YC is responsible for the conception, drafting, and revision of the thesis. All authors contributed to the article and approved the submitted version.

## Funding

This work was supported in part by the funds of the Collaborative Education Project of Industry-University Cooperation of the Ministry of Education of China (202102100053); the Key Talents Project of Maternal and Child Health Care in Jiangsu Province (SFY202103); and the Top Medical Expert Project of Tai Hu Ren Cai Plan (2021-9).

## Conflict of interest

The authors declare that the research was conducted in the absence of any commercial or financial relationships that could be construed as a potential conflict of interest.

## Publisher's note

All claims expressed in this article are solely those of the authors and do not necessarily represent those of their affiliated organizations, or those of the publisher, the editors and the reviewers. Any product that may be evaluated in this article, or claim that may be made by its manufacturer, is not guaranteed or endorsed by the publisher.
